# Will they lead by example? Assessment of vaccination rates and attitudes to human papilloma virus in millennial medical students

**DOI:** 10.1186/s12889-016-3969-x

**Published:** 2017-01-06

**Authors:** Nelia M. Afonso, Maurice J. Kavanagh, Stephanie M. Swanberg, Jeanne M. Schulte, Tracy Wunderlich, Victoria C. Lucia

**Affiliations:** 1 Department of Biomedical Sciences, Oakland University William Beaumont School of Medicine, 2200 North Squirrel Rd., Rochester, MI 48309 USA; 2 Clinical Skills Training & Simulation Center, Oakland University William Beaumont School of Medicine, 44300 Dequindre Rd., Sterling Heights, MI 48314 USA; 3 Department of Medical Education, Oakland University William Beaumont School of Medicine, 2200 North Squirrel Rd., Rochester, MI 48309 USA

**Keywords:** Human papillomavirus, Vaccination, Medical students, Attitudes, Needs assessment

## Abstract

**Background:**

Human papillomavirus (HPV) is the most common sexually transmitted infection in the United States. It is also well established that HPV viruses are responsible for a variety of cancers. Little is known about the prevailing knowledge and attitudes toward the HPV vaccine in our future healthcare providers, a majority of whom were among the first in the target age group to receive the vaccine; the same vaccine that they will in turn be expected to recommend to their patients. The aims of this pilot study were to examine the HPV vaccination rate among medical students and determine their knowledge about HPV and attitudes toward vaccination.

**Methods:**

To aid in the development of an HPV educational intervention, a needs assessment survey was administered to discover medical students’ knowledge and attitudes toward the HPV vaccine. All medical students at a Midwestern US medical school were invited to complete the survey.

**Results:**

Two hundred fourteen of 390 medical students completed the survey with 44% having been previously vaccinated. Although 82% of all respondents believed they would recommend the vaccine to family and friends, only 40% felt knowledgeable about the vaccine and 40% felt comfortable counseling patients. More positive attitudes and better knowledge scores were found in fully vaccinated students compared to non-vaccinated students. Provider recommendation was strongly associated with HPV vaccination status.

**Conclusions:**

This study revealed the unique perspectives of U.S. millennial medical students as the first group of future healthcare providers to have personally encountered the HPV vaccine. Overall, students’ knowledge as well as their comfort level in counseling patients was lacking. This assessment has guided the development of targeted educational interventions to address knowledge gaps and prepare students to appropriately discuss the vaccine with patients and parents and help protect young people from life threatening cancers.

## Background

Human papillomavirus (HPV) is the most common sexually transmitted infection in the United States [1]. The link between HPV viruses and malignancies in both men and women is well established with HPV genotypes 16 and 18 being responsible for nearly all cervical cancers [2]. HPV is also responsible for 91% of anal cancers, 75% of vaginal cancers, 63% of penile cancers, 69% of vulvar cancers and 60% of oropharyngeal cancers in the US [2, 3]. Three HPV vaccines are currently licensed in the United States for the prevention of infection due to HPV infection: Gardasil and Cervarix that offer protection against oncogenic genotypes 16 and 18 and the new Gardasil 9, which has the potential to prevent approximately 90% of cervical, vulvar, vaginal, and anal cancers caused by HPV types 16, 18, 31, 33, 45, 52, and 58 [4].

The United States Advisory Committee on Immunization Practices recommends vaccination for all girls and boys starting at 11 or 12 years of age, with catch up vaccination between ages 13–26 in women and 13–21 in men [5]. Even though it has been a decade since the HPV vaccine was approved in June 2006, there is still a lack of widespread uptake of this cancer-preventing vaccine. In 2014, only 40% of adolescent girls (age 13–17) and 22% of adolescent boys in the US received all 3 doses of the vaccine [6]. Due to the under-utilization of the HPV vaccination, 69 National Cancer Institute-designated Cancer Centers jointly issued a call to action to increase HPV vaccination rates in early 2016 [7].

Although well studied internationally [8, 9], very little is known about US medical students’ knowledge and attitudes about the HPV vaccine. This is especially important as US millennial medical students will be the first generation of providers who may have received the vaccine beginning in 2006; the same vaccine that they will now be expected to recommend to their patients. A 2016 study surveyed high school to health care professional students (including medical students) in New York State [10]. The authors partly conclude that this is related to health care professional students being less likely to have been recommended the vaccine by a health care provider than their younger counterparts - high school and college students [10]. Students who reported that their HPV vaccine information source was the doctor had a higher vaccine completion rate when compared to students whose information source was family and friends. However, one major limitation of this study was that data was not separated by type of health profession student, so prevailing attitudes of US medical students remains unclear. In their educational intervention for physicians, medical students, and non-physicians, Berenson et al. [11] found medical students had inadequate baseline knowledge of HPV epidemiology and the HPV vaccine, but after attending a brief, 30 minutes lecture increased their knowledge scores significantly. This demonstrates the potential for using educational interventions to increase HPV vaccine knowledge in this population.

### Objectives

The primary aims of this pilot study were to examine HPV vaccination rates among medical students and survey their knowledge and attitudes toward vaccination. Additionally, comparisons were sought between vaccinated versus non-vaccinated students. A secondary aim was to evaluate students’ perceived levels of comfort in counseling patients about HPV vaccine. This data will be used to develop and tailor educational interventions on the HPV vaccine at our medical school.

## Methods

### Participants

Participants included all 390 medical students enrolled at a single US Midwest allopathic medical school in October 2015. Participation in the study was voluntary with no compensation provided.

### Measurements

Participants were invited via email to complete an anonymous online survey on HPV. No signed consent was required since the survey was distributed via an online platform. An information sheet was emailed to the student to let them know about the study and that their computer IP address would not be collected. Participants could access the survey at a time and location of their choosing to optimize privacy while completing the survey. Completion of the survey indicated consent. The University’s Institutional Review Board approved the study protocol.

The email contained a link to an anonymous, 20-question online survey and an information sheet about the study. Two email reminders were sent 1 week apart. The survey included a mix of 5-point Likert scale, categorical (yes/no), and free text completion items. Items included: 1) demographic information; 2) vaccination status for HPV; 3) motivating factors for accepting or rejecting the vaccine; and 4) basic knowledge about HPV and the HPV vaccine. Attitude questions about HPV vaccination included: beliefs about the safety and efficacy of the vaccine, views on mandatory vaccination, comfort in providing counseling about vaccination, and intention to recommend vaccination to friends and family. Participants were also invited to comment on their views about the vaccine. Previous research on HPV and influenza vaccination uptake assisted in formulating the content of this pilot questionnaire [12–14].

### Qualitative analysis

Analysis of answers to the open-ended questions followed the grounded theory methodology [15]. A sub-group of investigators (JMS, TW and NMA) independently developed initial coding themes and then met to discuss and agree on a common coding approach and code definitions. Discrepancies were discussed and resolved. Responses were grouped under the key themes identified.

### Statistical analysis

Likert scale items were coded such that higher values corresponded to stronger agreement (1 = strongly disagree and 5 = strongly agree). Additionally, Likert scale items were collapsed to reflect overall agreement (by combining scores of 4 (agree) and 5 (strongly agree)). Categorical variables are reported as counts and percentage frequencies. They were examined using Fisher’s Exact test.

Sub-analyses were conducted based on gender and vaccination status. Students who had completed all 3 doses of the vaccine were considered fully vaccinated. Students who did not receive any doses of the vaccine were considered non-vaccinated. Partially vaccinated students (completed 1 or 2 doses of the vaccine) were excluded from the sub-analyses, as we did not have background information on the reasons for not completing all doses of the vaccine, which may introduce bias into the attitudes regarding the vaccine. All analyses used The SAS® System for Windows version 9.3, Cary, NC.

## Results

### Study sample

The overall response rate for the survey was 54.9% (214/390 students). Response rates varied by class, with 55.5% (71/128) of first year medical students, 62.5% (60/96) of second year medical students, 45.9% (45/98) of third year medical students, and 55.9% (38/68) of fourth year medical students responding to the survey. Demographic characteristics of respondents may be found in Table 1.

**Table 1:** Participant characteristics (*N* = 214)

Characteristics	*n* (%)
Age	
≤25 years	141 (66.2)
26–30 years	64 (30.0)
≥31 years	8 (3.8)
Gender	
Male	101 (47.4)
Female	112 (52.6)
Year in medical school	
1^st^ year	71 (33.2)
2^nd^ year	60 (28.0)
3^rd^ year	45 (21.0)
4^th^ year	38 (17.8)
Race/Ethnicity	
Asian	53 (25.0)
Black/African american	8 (3.8)
White	128 (60.4)
Hispanic/Latino	6 (2.8)
Other	17 (8.0)
Vaccination status	
Fully vaccinated (3 doses)	75 (35.2)
Partially vaccinated (2 doses)	11 (5.2)
Partially vaccinated (1 dose)	8 (3.8)
Non-vaccinated	119 (55.9)

### Knowledge of HPV vaccine

Although a majority of the 214 participants appeared well informed about HPV and vaccine safety (Table 2), there were evident gaps in knowledge about the protective effect of the HPV vaccine in cancers other than cervical, and immunity provided by the vaccine. Twenty-one percent of the participants (*n* = 44) did not know that the vaccine was recommended for girls and boys. Overall, fully vaccinated participants scored better in knowledge items than non-vaccinated participants (Table 3). Female participants were generally more knowledgeable than males, specifically with regards to the vaccine being recommended for both girls and boys (*p* = 0.002) (Table 2).

**Table 2:** Knowledge and Attitudes about HPV Vaccine by Sex (*N* = 214)

	Total sample positive response^a^ *N* = 214 n (%)	Male positive response^a^ *n* = 101 n (%)	Female positive response^a^ *n* = 112 n (%)	Fisher’s exact test *p*-value
Knowledge items				
HPV is a sexually transmitted infection	199 (93.4)	92 (92.0)	106 (94.6)	0.58
Men and women can be carriers of HPV	202 (94.8)	92 (91.1)	109 (98.2)	0.03*
HPV vaccine protects against genital warts	159 (74.3)	76 (75.2)	82 (73.2)	0.76
HPV vaccine protects against cervical cancer	195 (91.1)	91 (90.1)	103 (92.0)	0.64
HPV vaccine protects against other cancers	105 (49.5)	50 (50.0)	54 (48.6)	0.89
HPV vaccine is safe	191 (90.1)	87 (87.0)	103 (92.8)	0.17
HPV vaccine is effective	194 (91.1)	91 (90.1)	102 (91.9)	0.81
HPV vaccine has few side effects	163 (76.9)	69 (69.7)	93 (83.0)	0.03*
HPV vaccination leads to lasting immunity	120 (56.3)	56 (55.4)	63 (56.8)	0.89
HPV vaccine is recommended for girls and boys	169 (79.3)	70 (70.0)	98 (87.5)	0.002*
Attitude Items				
HPV vaccination may lead to risky sexual behavior	15 (7.0)	12 (11.9)	3 (2.7)	0.01*
I have enough information to be able to counsel about HPV vaccine	86 (40.3)	37 (37.0)	48 (42.9)	0.40
I feel comfortable counseling about HPV vaccination	86 (40.3)	40 (40.0)	45 (40.2)	1.00
I would recommend the HPV vaccine to friends and family	173 (81.6)	74 (74.8)	98 (87.5)	0.02*
HPV vaccine should be mandatory	112 (53.8)	47 (47.5)	65 (60.2)	0.07

^a^Positive response: Agree and Strongly Agree Likert scale responses were collapsed

*p < 0.05

**Table 3:** Knowledge and Attitudes about HPV Vaccine among Fully Vaccinated and Non-Vaccinated Medical Students

	Fully vaccinated, positive responses^a^ *n* = 75 *n* (%)	Non-vaccinated positive responses^a^ *n* = 119 *n* (%)	Fisher’s exact test *p*-value
Knowledge items			
HPV is a sexually transmitted infection	73 (97.3)	106 (89.8)	0.08
Men and women can be carriers of HPV	74 (98.7)	108 (91.5)	0.05
HPV vaccine protects against genital warts	58 (77.3)	86 (72.3)	0.50
HPV vaccine protects against cervical cancer	72 (96.0)	104 (87.4)	0.07
HPV vaccine protects against other cancers	36 (48.0)	56 (47.9)	1.00
HPV vaccine is safe	71 (95.9)	102 (85.7)	0.03*
HPV vaccine is effective	71 (95.9)	104 (87.4)	0.07
HPV vaccine has few side effects	64 (85.3)	83 (70.3)	0.02*
HPV vaccination leads to lasting immunity	49 (65.3)	58 (49.2)	0.04*
HPV vaccine is recommended for girls and boys	68 (90.7)	83 (70.3)	<0.001*
Attitude items			
HPV vaccination may lead to risky sexual behavior	4 (5.3)	10 (8.4)	0.57
I have enough information to be able to counsel about HPV vaccine	35 (46.7)	45 (38.1)	0.29
I feel comfortable counseling about HPV vaccination	34 (45.3)	44 (37.3)	0.29
I would recommend the HPV vaccine to friends and family	73 (97.3)	81 (69.2)	<0.001*
HPV vaccine should be mandatory	53 (72.6)	44 (38.3)	<0.001*

^a^Positive responses: Agree and Strongly Agree Likert scale responses were collapsed

*p < 0.05

### Attitudes toward HPV vaccine

While most participants believed they would recommend the vaccine to family and friend, less than half perceived themselves as having adequate information to counsel patients, or felt comfortable in their ability to counsel patients. Only a small percent (7%) perceived that the vaccine would lead to risky sexual behavior (Table 2). There were statistically significant differences (*p* < 0.05) in scores between vaccinated and non-vaccinated participants on perception of vaccine safety and efficacy (Table 3). Overall, over half of responding participants (53.8%, *n* = 112) felt that the vaccine should be mandatory (Table 2). However, a marked difference was seen in the view on mandatory vaccination between the fully vaccinated and non–vaccinated students (see Table 3), with 72.6% (*n* = 53) of fully vaccinated students agreeing that the vaccine should be mandatory as compared to 38.3% (*n* = 44) of non-vaccinated students (*p* < 0.001).

Of the 214 participants, 113 commented on “What are your views on HPV vaccination?” revealing five major themes: benefits of vaccination, emphasis on offering vaccine to boys and girls, moral implications associated with the HPV vaccine, need for counseling skills, and need for education. Frequency distribution of each theme and sample quotations that best represent each theme are provided in Table 4.

**Table 4:** Themes from open-ended question: What are your views on HPV vaccination? (*N* = 113)

Theme	Frequency *n* (%)^a^	Representative quotes
Benefits of vaccination	65 (57.5)	“From a public health stand point I feel everyone in the susceptible age range should get the HPV series because it really doesn’t harm anything, but it can be an effective way to prevent the spread of a preventable disease. It is harder to change society behavior than to arm people against the disease.”
Emphasis on offering vaccine to boys and girls	17 (15.0)	“promoting vaccination of males is essential. Many people, even fellow medical students, were unaware that males could and should receive the vaccination. I believe its so important because there is no test for men to get to know if they have HPV, and could unknowingly pass it on to many partners without ever finding out.”
		“Benefits outweigh the risks and it’s important for males to be vaccinated as well given the risk of infecting their partners.”
Moral implications associated with the HPV vaccine	15 (13.3)	“Though I would recommend my patients to receive the vaccine, I do not think it would be ethically wise to force it upon them”
		“… vaccine is geared to protection of HPV as it is acquired sexually, therefore imposing this would assume that the patient is or will be sexually active in the near future. This may interfere with their personal and spiritual beliefs and thus making such assumptions would be inappropriate….”
		“I really think HPV and all vaccinations should be strongly encouraged but I am on the fence about mandating them. I think mandates make people hostile and more likely to resist and feel forced into decisions that they aren’t comfortable with. From a public health perspective I think getting folks on board with HPV vaccination is a big deal, especially since now we are seeing other cancers (not just cervical) related to it..”.
Need for counseling skills	8 (7.1)	“I think we need to educate providers about the best ways to provide the information to not make it sound like an STD vaccine.”
		“When I got it Gardasil had just come out and then I moved so I never got the third one; I wish my doctor had counseled me further (I don’t remember being counseled, just told by her to get it)”
Need for education	10 (8.8)	“To be honest, I never knew much about HPV vaccinations or the recommendations for it, just that it existed.”
		“Do not know enough about it to really have a view!”

^a^51% of reflections contained multiple themes (range 2–3)

### Vaccination status

Fully vaccinated (*n* = 75) and partially vaccinated (*n* = 19) students accounted for 44.1% (*n* = 94/213) of the sample. The 25 years and below age group had the highest number of participants who had received at least one dose of vaccine (50.7%, *n* = 71/140), when compared to both the 26–30 years age group (32.8%, *n* = 21/64) and 31 years and above age group (25.0%, *n* = 2/8). In this youngest age group, 59.0% (*n* = 49/83) of women received the complete series of the vaccine as compared to 8.8% (*n* = 5/57) of men; 72.3% (*n* = 60) of women and 19.3% (*n* = 11) of men in this group received at least one dose.

The principal reasons for vaccination cited by participants who had received at least one dose of the HPV vaccine were provider recommendation (71.3%, *n* = 67) and desire for self-protection (67.0%, *n* = 63). Among the non-vaccinated group, the main reasons identified were lack of provider recommendation (48.7%, *n* = 58), a perception that they were not at risk for HPV (22.7%, *n* = 27), and lack of information about the vaccine (18.5%, *n* = 22). Parental desire for vaccination played a role for 48.9% (*n* = 46) respondents receiving at least one dose of the vaccine, whereas parental reluctance was a 8.4% (*n*=10) of non-vaccinated respondents. Additionally, 21.8% (*n* = 26) of non-vaccinated participants believed they were not in the right age group to be vaccinated. The fully vaccinated participants also felt they would recommend the vaccine to friends and family compared to non-vaccinated participants (*p* ≤ 0.01) (Table 3).

## Discussion

The HPV vaccine was introduced in 2006 for girls and added to the list of approved vaccines for boys in 2011. Although several studies have examined vaccination practices and attitudes toward HPV vaccination in practicing physicians [14, 16–18] and in international medical students and residents [8, 9, 11–13, 19], the authors only found one study reporting HPV vaccination rates in US medical students [10]. However, in that study all health care professional students were lumped into a single student category so there was no way to separate out medical student data [10]. Assessing medical student knowledge and attitudes about the HPV vaccine is vital since many in this group were likely to be the initial recipients of the HPV vaccine that was first offered in 2006. When these students become physicians they will carry with them this unique personal experience not found in other older health care providers. The current study represents the first report of HPV vaccination rates, knowledge, and attitudes among these US medical students.

In the under 25 age group, we found HPV vaccination rates for female and male medical students who had received at least one dose to be 72.3% and 19.3% respectively, a markedly higher coverage rate than the most recently reported national figures of 40.2% for females and 8.2% for males in a similar age group in 2014 [20]. Similar to other college students, the higher overall vaccination rates among medical students may be the result of better knowledge, motivation, and better access to vaccinations and healthcare [21–23]. The majority of the students who were vaccinated appeared to have a better understanding of HPV pathogenesis and the safety and efficacy of the HPV vaccine. It is additionally interesting to note that 73% of vaccinated students felt that vaccination should be mandatory as compared to 38% of non-vaccinated students. Although the authors are aware of no data on HPV vaccine receipt by health care providers, we believe that positive attitudinal differences in medical students who are HPV vaccine recipients, are similar to other vaccine studies, which show previous vaccination tends to impart a positive influence [13, 24]. Similar to our previous work on influenza vaccine, the current study showed a significantly higher likelihood of previously vaccinated students recommending influenza vaccination to friends and family [13].

The current study strongly supports the assertion that provider recommendation is a consistent and powerful predictor of vaccination, as evidenced by the large proportion of vaccinated students citing provider recommendation as a reason they received it. Gilkey et al. [16] found that participants who received a provider recommendation were 35 times more likely to receive HPV vaccination [25]. Gilkey et al. [16] also found that vaccine uptake was adversely affected if health providers introduced the topic of HPV vaccination without expressly recommending it. Again, when comparing health professions students to high school and college level, Suryadevara et al. [10] found that most health professions students had not been counseled about the HPV vaccine and attributed this to lower uptake of the vaccine by this population.

Our survey also revealed deficits in student knowledge and misperceptions about HPV. Although a majority of the students were well informed about HPV transmission (93.4%), cervical cancer (91.1%), and vaccine safety (90.1%), there was lack of knowledge about the protective effect of the HPV vaccine in cancers other than cervical (49.5%) as well as the duration of immunity provided by the vaccine. (56.3%) There was also the perception among the non-vaccinated students that they were not at risk for HPV. Greater emphasis, therefore, needs to be placed in teaching about the importance of HPV in disease etiology and the causal role of HPV in a variety of cancers [2, 3]. Initiatives geared toward educating medical students about HPV vaccine should focus on cancer prevention and include data on safety and efficacy and strategies for positively framing counseling messages to patients.

Our previous experience illustrates the effectiveness of an early intervention coupled with experiential learning when teaching about influenza vaccination [13]. Guided by this assessment, beginning this academic year, we have developed and integrated multi-faceted educational interventions aimed at improving knowledge of HPV, cancer and vaccines, as well as providing students hands on experience practicing vaccine counseling with standardized patients. We anticipate these will play a pivotal role in the endorsement of this vaccine by our future physicians. In the short term these skills will also assist students in discussing the importance of this vaccine during their community engagement activities. Further studies to evaluate the impact of these teaching strategies aimed at millennial medical students are currently being planned.

### Limitations of the study

This study has several limitations. First, this study was conducted at a single medical school on a relatively small sample size that may limit generalizability. Although national survey data may have revealed regional variations, there are no indications that this institution’s students are noticeably different from those in other US allopathic medical schools. Self-reported data made it difficult to verify the accuracy of respondents’ vaccination status and we did not assess age at initiation of the vaccine. Also, this study did not seek to compare medical student attitudes between HPV and other vaccines. The survey was not designed to assess where the students obtained their knowledge and how it may have impacted their beliefs. Finally, the survey may have been influenced by a response bias – between those who responded to the email survey and those who opted not to participate, which prevented us from identifying possible patterns of characteristics among non-respondents.

## Conclusions

Medical students are a key audience for HPV-related communication and training not only because of their impending role as healthcare providers, but also as future policy makers. We believe a particularly important contribution made by this study is the perspective of millennial medical students as a unique group of future healthcare providers, who will be the first to have had a personal experience with the vaccine. This study also highlights a pervasive lack of understanding regarding the protection against cancer other than cervical, conferred by the HPV vaccine. Although students acknowledge the importance of the vaccine and the need for patient education and counseling to prevent HPV-related cancers through vaccine compliance, they nonetheless feel ill prepared to provide that counseling. Students should be taught age-relevant approaches to counseling parents, adolescents, and young adults about this cancer-preventing vaccine that could save the lives of millions.

It is hoped that medical student personal experiences with the vaccine, supplemented by medical school education about HPV and appropriate vaccine-counseling skills, will allow them to share information with patients and parents in a clear, reassuring way, devoid of stigma. We anticipate they will be healthcare providers who play a critical role in recommending the HPV vaccine and help protect young people from life threatening cancers.
